# Fundamental Properties of Expanded Perlite Aggregated Foamed Concrete with Different Supplementary Cementitious Materials

**DOI:** 10.3390/ma18122671

**Published:** 2025-06-06

**Authors:** Kaixing Fan, Jie Wei, Chengdong Feng

**Affiliations:** 1 School of Architecture, Xi’an University of Architecture and Technology, Xi’an 710055, China; fankaixing@xauat.edu.cn; 2 Tianjin Key Laboratory of Prefabricated Buildings and Intelligent Construction, School of Civil and Transportation Engineering, Hebei University of Technology, Tianjin 300401, China

**Keywords:** foamed concrete, expanded perlite (EP), supplementary cementitious materials (SCMs), material performance

## Abstract

This study investigates the effects of supplementary cementitious materials (SCMs) on the material performance of foamed concrete containing lightweight coarse aggregates, namely hydrophobically modified expanded perlite (EP). The EP aggregates were treated with a sodium methyl silicate solution to impart water-repellent properties prior to being incorporated into the foamed concrete mixtures. Ordinary Portland cement (OPC) was partially replaced with various SCMs, namely, silica fume (SF), mineral powder (MP), and metakaolin (MK) at substitution levels of 3%, 6%, and 9%. Key indicators to evaluate the material performance of foamed concrete included 28-day uniaxial compressive strength, thermal conductivity, mass loss rate under thermal cycling, volumetric water absorption, and shrinkage. The results noted that all three SCMs improved the uniaxial compressive strength of foamed concrete, with MP achieving the greatest improvement, approximately 97% at the 9% replacement level. Thermal conductivity increased slightly with the addition of SF or MP but decreased with MK, highlighting the superior insulation capability of MK. Both SF and MK reduced the mass loss rate under thermal cycling, with SF exhibiting the highest thermal stability. Furthermore, MK was most effective in minimizing water absorption and shrinkage, attributed to its high pozzolanic reactivity and the resulting refinement of the microstructures.

## 1. Introduction

Rapid urbanization has intensified resource consumption and environmental pollution [1,2,3]. The construction industry accounts for approximately 40% of global energy use and carbon emissions, primarily from building operations [4,5,6]. Improving the thermal performance of building envelopes, especially via external wall insulation, is critical for reducing operational energy consumption [7]. Accordingly, there is growing emphasis on developing sustainable, energy-efficient insulation materials for residential applications to meet global low-carbon targets [8,9,10].

Lightweight foamed concrete has become an increasingly attractive choice for sustainable and energy-efficient exterior wall insulation, offering superior durability and low thermal conductivity relative to conventional expanded polystyrene board systems [11,12,13,14]. Fabrication techniques could be divided into two primary categories: chemical foaming, which involves the addition of reactive foaming agents to the cementitious mixture that release gas through exothermic reactions, and physical foaming, which relies on high-speed mechanical agitation to entrain air and produce stable foam prior to mixing with the cement slurry. By adjusting the volume of foam, the density of foamed concrete can be precisely controlled to meet specific design requirements [15]. This density variation exerts a strong influence on key properties: lower densities generally improve thermal insulation but also lead to a reduction in compressive strength [16,17,18].

Optimizing the selection of lightweight coarse aggregates is essential for improving both compressive strength and structural stability in foamed concrete while maintaining its low-density advantage. Organic polymer insulation materials such as expanded polystyrene, extruded polystyrene, polyurethane foam, and rubber sponge have been extensively studied because they offer low thermal conductivity, excellent hydrophobicity, and ease of molding [19,20,21,22,23]. However, these materials are prone to flammability, shrinkage, and melting at elevated temperatures, raising environmental concerns. In fire scenarios, they may release harmful combustion products that accelerate flame propagation and jeopardize both human safety and property [24,25,26,27]. To overcome these limitations, attention has shifted to inorganic aggregates with inherent flame-resistant properties. Expanded perlite, an abundant acidic volcanic mineral, offers a combination of low bulk density, superior thermal insulation, excellent environmental compatibility, and robust fire resistance. Its application as the primary coarse aggregate in foamed concrete has significantly improved both mechanical performance and thermal insulation effectiveness [28,29,30,31,32].

Expanded perlite is characterized by a naturally porous, honeycomb-like microstructure that endows it with high water absorption capacity [33]. However, this characteristic degrades its thermal insulation performance, primarily because the thermal conductivity of water is approximately 23 times greater than that of air. When water occupies the internal voids of expanded perlite, the pores act as thermal bridges, substantially reducing or entirely negating the insulating effect [34,35]. To address this issue, recent research has explored a range of functional modifications at both the surface and structural levels. Li et al. [36] applied a hydrophobic membrane coating to expanded perlite, altering its surface chemistry to reduce water uptake while maintaining thermal insulation. Mehmet et al. [37] utilized radio frequency plasma discharge to deposit poly (hexafluoro butyl acrylate) films on the perlite surface, which conferred super-hydrophobicity and reduced water holding capacity from 70% to approximately 4%. Jia et al. [38] introduced aerogel into the pore network of perlite to create a composite insulation material; this approach not only improved chemical stability and water resistance but also achieved a 30% reduction in thermal conductivity compared with untreated expanded perlite.

Currently, foamed concrete primarily uses ordinary Portland cement as the primary binder system. However, the manufacture of ordinary Portland cement is highly energy-intensive and generates significant carbon emissions, posing serious environmental challenges and impeding progress toward sustainability targets [39]. To mitigate these impacts, industrial byproducts rich in aluminum and silicon ions, such as fly ash, ground granulated blast furnace slag, and silica fume, have been adopted as supplementary cementitious materials. These materials can partially replace ordinary Portland cement in foamed concrete, thereby offering a more environmentally responsible approach without compromising mechanical performance [40,41,42]. For examples, Gong et al. [43] demonstrated that a blend containing 30% slag and 6% silica fume produced foamed concrete with reduced porosity, improved frost resistance and markedly enhanced strength and durability. Chen et al. [44] noted that adding silica fume accelerated early-age strength development during ordinary Portland cement hydration. Zhang et al. [45] found that increasing slag content from 0% to 16% improved both the uniaxial compressive and flexural strength of foamed concrete. Although these studies have thoroughly characterized the mechanical and durability benefits of supplementary cementitious materials incorporation, research into their influence on the thermal conductivity of foamed concrete remains scarce, highlighting the need for further investigation in this area.

This study pioneered a strategy for optimizing sustainable foamed concrete by synergistically integrating chemically modified lightweight aggregates with multi-component SCM blends, addressing durability and mechanical characteristics in thermal materials. In the present study, expanded perlite was rendered hydrophobic by surface treatment with a sodium methyl silicate solution, after which the modified aggregate was employed as a lightweight coarse component in foamed concrete. The concrete matrix was prepared using ordinary Portland cement and a chemical foaming agent. To improve both sustainability and material performance, silica fume, mineral powder, and metakaolin were each incorporated as supplementary cementitious materials at mass replacement levels of 3%, 6%, and 9% of the cement content. To validate the influence of aggregate modification and cement replacement on the mechanical and durability characteristics of foamed concrete under a range of simulated service conditions, a comprehensive suite of performance tests was conducted, including 28-day uniaxial compressive strength, thermal conductivity, mass loss rate following repeated thermal cycling, volumetric water absorption, and drying shrinkage.

## 2. Materials and Methods

### 2.1. Raw Materials

**Figure 1:**
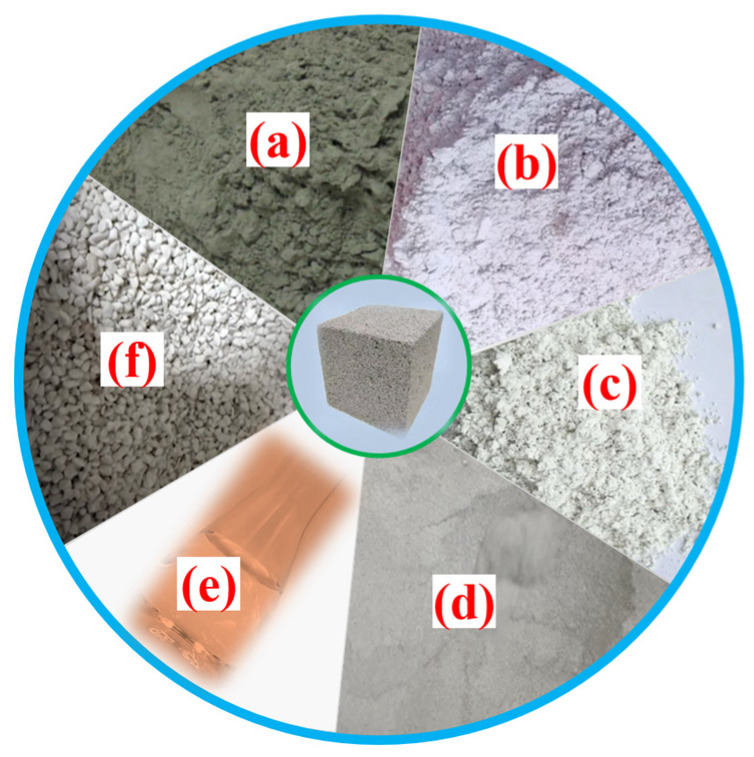
Raw materials adopted for foamed concrete (image by the authors): (a) OPC (b) MP, (c) MK, (d) SF, (e) foaming agent, and (f) EP.

**Figure 2:**
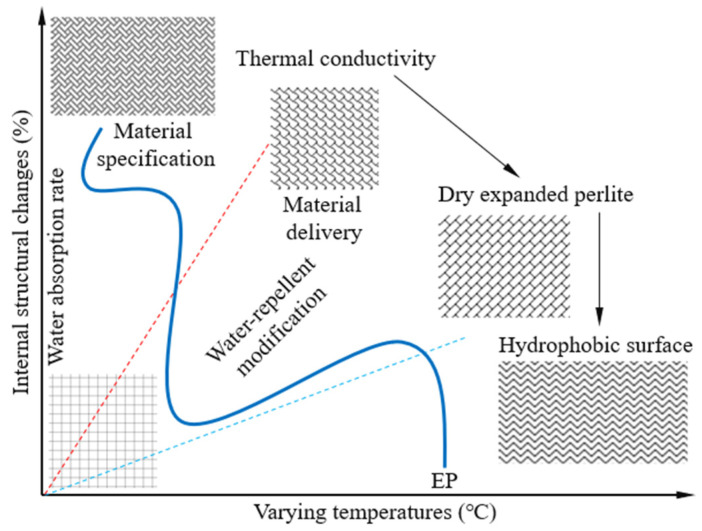
Schematic diagram for internal microstructure changes in EP under varying temperatures.

The foamed concrete developed in this study was primarily composed of binder materials, lightweight coarse aggregates, and a chemical foaming agent, as illustrated in Figure 1. The binder system comprised ordinary Portland cement (OPC), mineral powder (MP), metakaolin (MK), and silica fume (SF). The materials were procured from Hubei RockTek Co., Ltd., (Daye City, China) Specifically, the OPC used was P.O. 42.5-grade; the MP was of S95-grade ground granulated blast furnace slag; the MK was classified as Type II with standard pozzolanic activity, and the SF met the specifications for Grade II. The chemical compositions and physical properties of MP, MK, and SF are presented in Table 1. The chemical foaming agent utilized was a hydrogen peroxide solution with a mass fraction of 30% and a purity of 99.5%. The expanded perlite (EP), with particle sizes ranging from 3 mm to 5 mm, served as the lightweight coarse aggregate. To enhance its water resistance, the EP was surface-modified by immersion in a sodium methyl silicate solution. Sodium methyl silicate is an organosilicon-based water repellent that, when dissolved in water under alkaline conditions, undergoes hydrolysis to produce a dense hydrophobic coating. The efficiency of this hydrophobic treatment has been extensively validated in the literature [46,47]. Following treatment, the EP is believed to maintain its inherent loose and porous honeycomb microstructure, although structural variations may occur under temperature cycling. As depicted in Figure 2, it is hypothesized that the EP contributes to the formation of the skeletal framework of the foamed concrete, which may enhance its mechanical strength and structural stability. Additionally, EP is expected to play a significant role in improving the thermal insulation performance of the cementitious composites.

### 2.2. Sample Preparation

**Figure 3:**
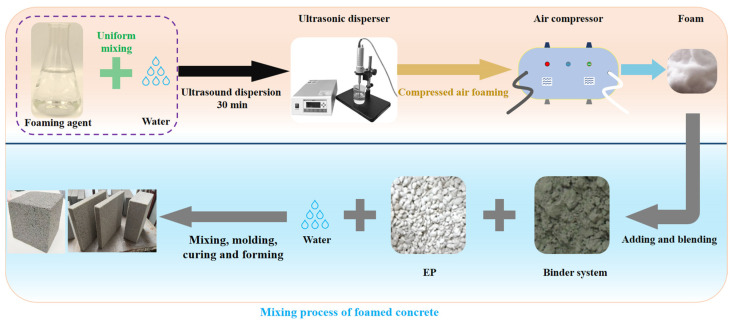
Process of foamed concrete samples preparation.

The detailed mixture proportions of the foamed concrete are summarized in Table 2. In this study, three levels of supplementary cementitious material (SCM) substitution, namely, 3%, 6%, and 9%, were investigated. The SCMs were MP, MK, and SF, each used as a partial substitute for OPC. The water-repellent modified EP was employed as the lightweight coarse aggregate. The foamed concrete samples were produced through the prefabricated foam method and mixing process, as illustrated in Figure 3. The foam solution was prepared by mixing the foaming agent, stabilizer, and water, followed by ultrasonic dispersion for 30 min prior to use. To ensure dimensional stability and optimize performance, the demolded samples were subjected to water curing under controlled conditions, i.e., 20 ± 5 °C and relative humidity no less than 90%. The mechanical and thermal performance evaluations were carried out following a standard 28-day curing period.

### 2.3. Testing Methods

**Figure 4:**
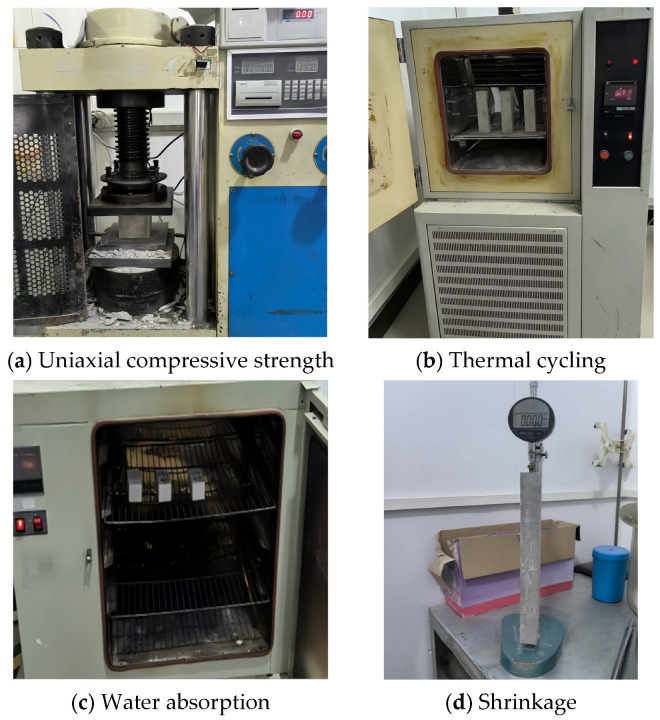
Experimental setup of material performance tests for foamed concrete samples.

All material tests were commissioned by Shaanxi Building Materials Research Institute Co., Ltd., (Xi’an City, Shaanxi Province, China). The uniaxial compressive strength of the foamed concrete samples was determined in accordance with the GB/T 50081:2019 [48], using a JES-2000A compression testing machine, with the experimental setup presented in Figure 4a. Cubic samples with a side length of 100 mm were subjected to axial loading at a constant rate of 0.3 MPa/s to 0.5 MPa/s until failure. For each mixture group, five samples were tested, and the average value was reported as the 28-day uniaxial compressive strength.

The thermal conductivity of the foamed concrete was measured employing a dedicated thermal conductivity device (IMKD-A), following the specifications outlined in GB/T 10295:2008 [49]. Three plate-shaped samples, each measuring 300 mm (length) × 300 mm (width) × 30 mm (thickness), were cured under standard conditions for 28 days and subsequently dried to a constant weight. The thermal conductivity was calculated as the average value obtained from the three identical samples, with a measurement precision up to 0.00001 W/(m·K).

Thermal cycling tests were conducted using prismatic samples with dimensions of 100 mm × 100 mm × 300 mm to assess the durability of foamed concrete exposed to temperature cycling. The tests were carried out in a programmable high–low temperature chamber (WGD7005), as illustrated in Figure 4b, with a controlled temperature range from −18 °C to 50 °C and heating/cooling rates not exceeding 10 °C/min. Thermal cycles were applied in increments of 0, 30, 60, 90, and 120 cycles, with each cycle consisting of 6 h at 50 °C, followed by 6 h at −18 °C, yielding a 12 h full cycle. Prior to testing, the samples were cured for 28 days and subsequently conditioned at a temperature of 20 ± 2 °C and a relative humidity of 95 ± 1% for 7 days to achieve a saturated surface-dry condition. Testing was terminated once the mass loss rate exceeded 5% or visible through-cracks appeared, after which performance evaluations were promptly conducted. The mass loss rate ($\text{∆}m_{n}$) of the tested sample after *n* thermal cycles was computed using the following equation:

(1) $$\text{∆}m_{n}=\frac{m_{0}-m_{n}}{m_{0}}\;\text{×}\;100\%$$

where, $m_{0}$ is the initial mass of the sample before thermal cycling, and $m_{n}$ is the mass of the sample after *n* thermal cycles.

The water absorption capacity of foamed concrete was tested using the DHG-9146A experimental apparatus shown in Figure 4c, in accordance with the GB/T 17146:2015 [50]. Three oven-dried prismatic samples with dimensions of 50 mm × 50 mm × 70 mm were fully submerged in a water tank maintained at 20 ± 5 °C. The water level was kept 30 mm above the top surface of the samples to ensure full immersion. After 48 h of immersion, surface moisture was gently removed using a dry cloth, and the saturated mass was recorded. The water absorption rate ($W_{a}$) was calculated employing the following equation:

(2) $$W_{a}=\frac{m_{w}-m_{d}}{m_{d}}\;\text{×}\;100\%$$

where $m_{w}$ is the saturated surface-dry mass of the sample, and $m_{d}$ is the dry mass of the sample.

The shrinkage testing of the foamed concrete was conducted in compliance with the GB/T 50082:2009 [51], employing a BC156-300 length comparator, wherein the test setup can be seen in Figure 4d. For each mixture group, three prismatic samples measuring 25 mm × 25 mm × 200 mm were prepared. After 24 h, the samples were demolded and subjected to standard curing for an additional 48 h. Subsequently, the samples were transferred to a controlled drying environment maintained at 20 ± 2 °C, where length changes were monitored over a 90-day period. The shrinkage strain of the foamed concrete samples at time *t* was calculated using the following equation:

(3) $$\epsilon_{\mathrm{st}}=\frac{\left(L_{b}-{\;L}_{t}\right)}{L_{0}}\;\text{×}\;1000\;\text{×}\;100\%$$

where $\epsilon_{\mathrm{st}}$ represents the shrinkage strain (10^−6^) at time *t*; $L_{b}$ denotes the initial length of the sample before drying; $L_{t}$ indicates the measured length of the sample at time *t*; $L_{0}$ refers to the gauge length of the sample, which is typically set at 450 mm for standard prismatic samples.

## 3. Results and Discussion

### 3.1. Uniaxial Compressive Strength

**Figure 5:**
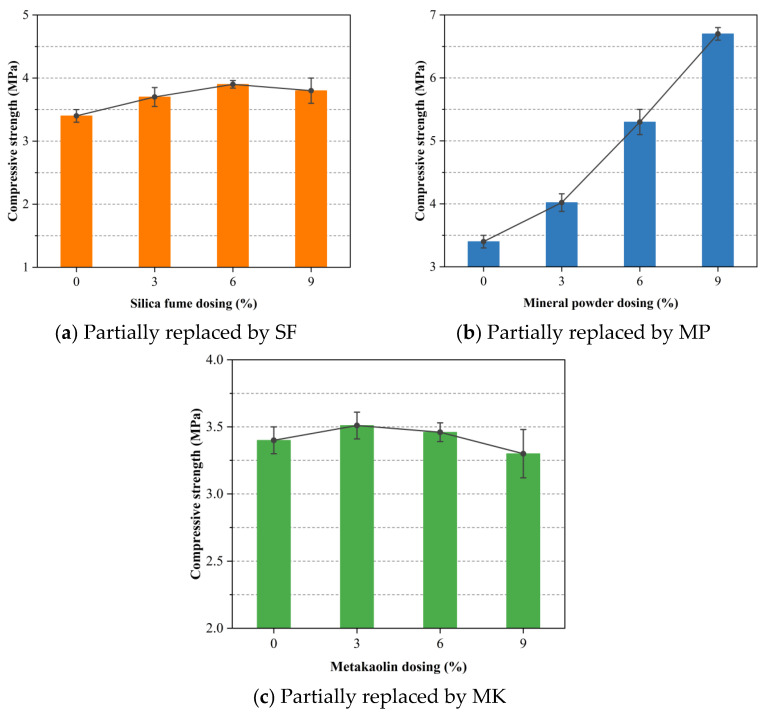
Effect of SCM dosage on the uniaxial compressive strength of foamed concrete with mass-equivalent cement replacement.

Figure 5 illustrates the effects of SCM dosage on the uniaxial compressive strength of foamed concrete under mass-equivalent cement replacement of OPC. As shown in Figure 5a, the addition of SF first enhanced and then slightly reduced compressive strength as SF content increased. The highest strength of 3.9 MPa was observed at a 6% replacement level, representing a 14.7% gain compared to the control mixture without SF. At 9% SF, strength decreased marginally but remained above that of the control, indicating that moderate SF levels optimize performance. The strength gain is attributed to the low density of SF, which under mass-equivalent substitution increases the proportion of cementitious material and reduces foam volume. Moreover, the pozzolanic reaction between reactive SiO_2_ in SF and the Ca(OH)_2_ generated during hydration produces secondary calcium silicate hydrate (C-S-H) with a lower (Ca/Si) ratio, thereby refining the microstructure [45,52]. Excessive SF, however, can diminish the effective cementitious fraction and impair slurry flow, leading to uneven foam distribution and reduced strength.

Figure 5b shows a pronounced improvement in compressive strength with increased MP content, reaching a peak of 6.7 MPa at a 9% replacement rate. This enhancement results from the participation of MP in secondary hydration reactions that yield dense gel phases, strengthening interparticle bonds and the overall matrix [53]. The ultrafine MP particles also act as micro-fillers, occupying capillary voids, reducing both macro- and interconnected pores and thereby improving load-bearing capacity [54]. Furthermore, MP promotes the formation of a uniform pore structure by inhibiting bubble coalescence during foaming, which facilitates even stress distribution under compression. Among the SCMs tested, the superior strength enhancement observed with MP may be attributed to its more stable and sustained hydration process, which continuously contributes to strength development throughout the 28-day curing period.

As depicted in Figure 5c, MK addition exerted a beneficial effect on compressive strength up to a 3% replacement level, where the maximum strength of 3.51 MPa was recorded. The fine particle size of MK enhances packing density around OPC grains, particularly within the interfacial transition zone (ITZ), and its pozzolanic and microaggregate effects promote the development of dense hydration products [55,56]. Beyond the optimal 3% substitution, strength declined, which may be explained by the formation of less dense calcium aluminosilicate hydrate (C-A-S-H) phases and the reduction in reactive cement content. Additionally, unreacted MK particles can remain dispersed within the matrix, hindering further densification and thus limiting strength development.

According to previous studies [57], the compressive strength of foamed concrete is closely related to its bulk density. Table 3 presents the bulk density values of the specimens for each mix group in this study. The increase in SF content can enhance the matrix densification, thereby promoting strength development. However, an excessive amount of silica fume may interfere with the foaming process, potentially offsetting these strength gains. Likewise, the incorporation of MP increased the bulk density of foamed concrete, and its strong influence on strength enhancement was clearly evident. MK may contribute to improved foam stability during both the mixing and hardening processes, allowing for greater retention of air voids. As a result, at lower replacement levels, the density of foamed concrete decreases while its compressive strength is maintained or slightly improved.

### 3.2. Thermal Conductivity

**Figure 6:**
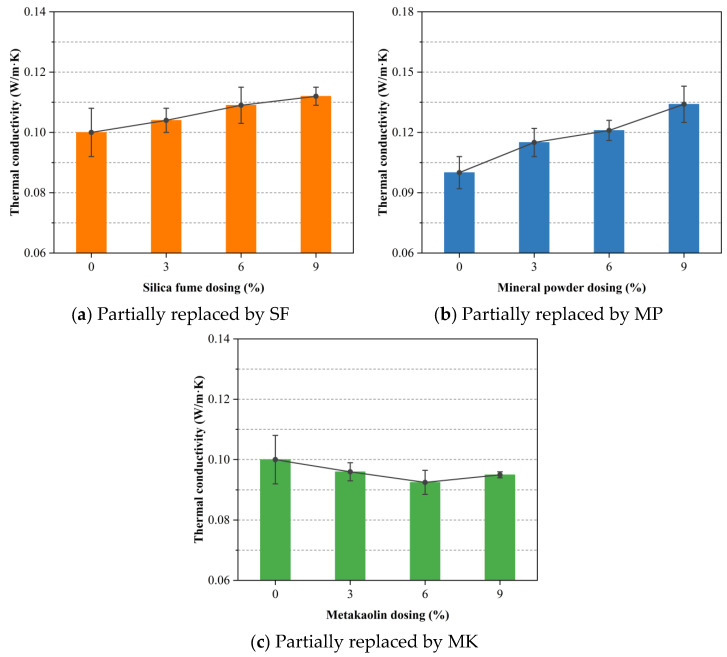
Effect of SCM dosage on the thermal conductivity of foamed concrete with mass-equivalent cement replacement.

Thermal conductivity, which measures the rate of heat flow per unit thickness under a given temperature gradient, is a fundamental property for evaluating the insulation performance of concrete. Figure 6a shows that replacing OPC with increasing amounts of SF from 0 to 9% led to a slight rise in thermal conductivity, from 0.100 to 0.112 W/(m·K). This increase is explained by the ultra-fine SF particles filling micro-voids in the matrix, thus increasing bulk density and increasing heat conduction marginally. Despite this densification, pore-structure refinement afforded by SF maintains the low connectivity of air-filled voids, ensuring that the overall insulation performance remains high [58,59].

As shown in Figure 6b, the addition of MP similarly produces a gradual increase in thermal conductivity with higher substitution levels. The delayed pozzolanic reaction of MP compared with cement hydration extends setting time, which can promote foam collapse and the interconnectivity of pores [59]. An increase in interconnected porosity provides more continuous pathways for heat flow, explaining the observed rise in thermal conductivity. Lu-Shu et al. [60] experimentally demonstrated that the thermal conductivity of concrete increases with its density. Similarly to SF, MP exhibits a pronounced micro-filler effect, which may enhance particle packing and increases the proportion of the solid phase within the concrete matrix. This densification of the internal structure was likely responsible for the slight increase in thermal conductivity observed with the incorporation of SF and MP.

Figure 6c demonstrates that MK consistently reduces thermal conductivity across all tested replacement levels. This aligns with the observed gradual decrease in the bulk density of the foamed concrete. A minimum value occurs at 6 percent MK, indicating an optimal blend of matrix refinement and insulation. The pozzolanic reaction of MK, involving SiO_2_ and Al_2_O_3_ with calcium hydroxide from hydration, produces additional amorphous C-S-H gel. These gels exhibit lower intrinsic thermal conductivity and form a denser, closed-pore network that impedes heat transfer. Beyond the 6% threshold, however, excessive MK hinders workability and foam stability, which slightly increase thermal conductivity.

### 3.3. Durability Under Thermal Cycling

**Figure 7:**
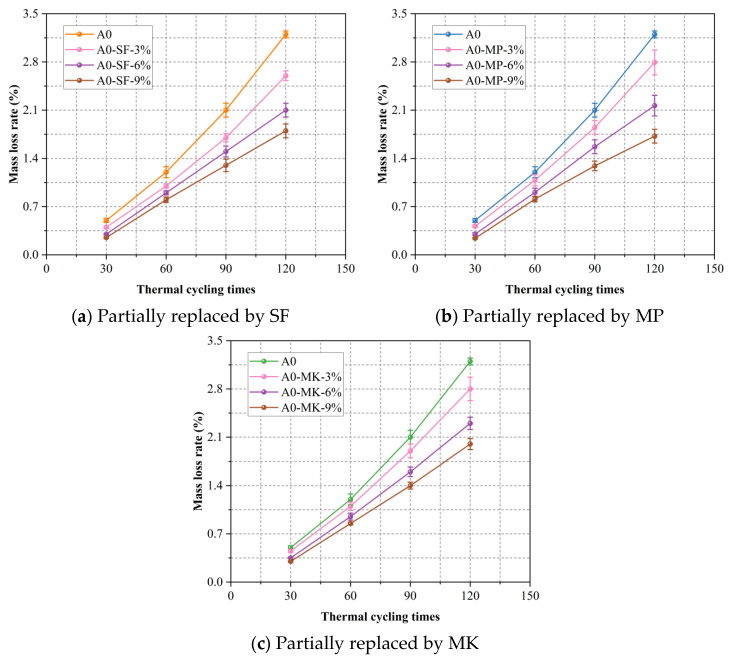
Effect of SCM dosage on the mass loss rate of foamed concrete under thermal cycling with mass-equivalent cement replacement.

Thermal cycling tests were conducted to evaluate the durability of foamed concrete subjected to extreme temperature fluctuations. Specimens underwent 30, 60, 90, and 120 cycles of alternating exposure, and mass loss was recorded after each interval. As shown in Figure 7, all sample groups experienced increasing mass loss with additional thermal cycles. However, foamed concrete mixtures incorporating SCMs, such as SF, MP, or MK, exhibited substantially lower mass loss rates than the control mixture A0. Higher SCM replacement levels further enhanced resistance to thermal degradation. After 120 cycles, the A0 control lost 3.2% of its mass, whereas the A0-SF-9%, A0-MP-9%, and A0-MK-9% mixtures lost only 1.8%, 1.72%, and 2.0% of their mass, corresponding to relative reductions of 43.8%, 46.3%, and 37.5%, respectively. These findings are consistent with the existing literature. For instance, Duan et al. [61] reported that SF reduced mass loss under thermal cycling by enhancing microstructure and thermal resistance. It is indicated that SCMs improve interfacial bonding between aggregates and the cement matrix, increase resistance to crack initiation, and slow the propagation of microcracks induced by repeated expansion and contraction. The fine SCM particles also act as micro-fillers to refine the pore network, while their pozzolanic reactions generate additional, chemically stable gel phases that maintain matrix integrity and reduce surface spalling or powdering. Notably, the SF-modified mixtures at 3% and 6% replacement levels demonstrated the best performance under thermal cycling, though the 9% MP mixture delivered the greatest enhancement in mass retention.

### 3.4. Volumetric Water Absorption

**Figure 8:**
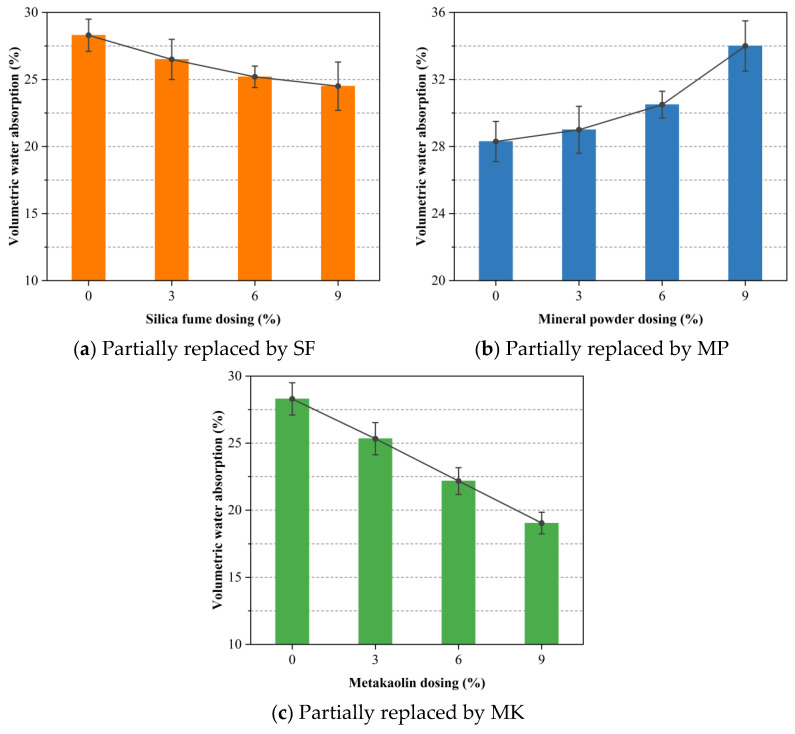
Effect of SCM dosage on the volumetric water absorption of foamed concrete with mass-equivalent cement replacement.

Volumetric water absorption serves as a crucial indicator of pore structure compactness and connectivity in foamed concrete, reflecting its high intrinsic porosity. To assess the influence of SCMs on this property, 28-day cured specimens incorporating SF, MP, and MK as partial mass-equivalent replacements for OPC were evaluated, as shown in Figure 8. Figure 8a demonstrates that increasing SF content induces a steady reduction in volumetric water absorption. At a 9% SF substitution level, water absorption declined to 24.5%, representing a reduction of approximately 13.4% compared to the control sample. This improvement stems from the high pozzolanic reactivity of SF, which consumes calcium hydroxide to form additional C-S-H that fills capillary pores and densifies the microstructure. Related studies have also found that the addition of SF can reduce the water absorption of foamed concrete, which is associated with decreased pore connectivity [39]. Moreover, SF refines the interfacial transition zone between aggregate and paste, thereby minimizing weak interfaces prone to water ingress and improving impermeability.

The volumetric water absorption of foamed concrete continuously decreased with increasing MK content, as shown in Figure 8c. At a 9% replacement level, the volumetric water absorption dropped to 19.04%, representing a reduction of approximately 32.7% compared to the control group. This improvement is possibly attributed to the high pozzolanic reactivity of MK. Despite the overall reduction in bulk density, MK still contributes to microstructural refinement by filling micro-voids and reducing pore connectivity, thereby enhancing the impermeability of the matrix [62].

Conversely, Figure 8b reveals that increasing MP substitution results in higher volumetric water absorption. This trend is mainly due to the lower pozzolanic reactivity of MP relative to OPC, which slows the formation of pore-filling hydration products. Furthermore, high MP content can negatively affect the fresh-state rheology of the slurry and compromise foam stability during mixing and curing. Such disturbances in foam retention and surface tension control may lead to irregular pore architectures, including the collapse or coalescence of air voids. These microstructural irregularities create continuous water pathways that exacerbate absorption and diminish the overall impermeability.

### 3.5. Shrinkage

**Figure 9:**
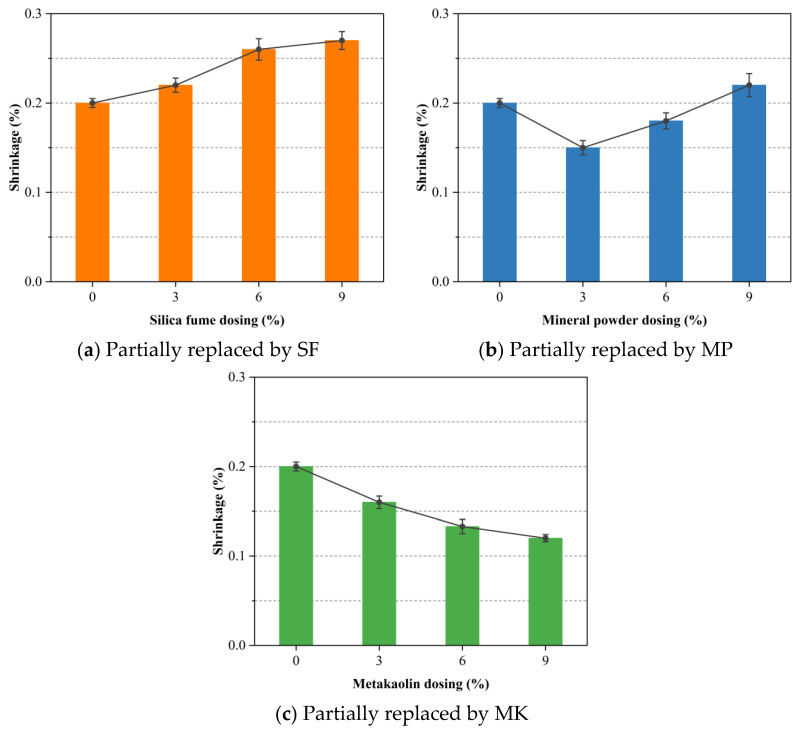
Effect of SCM dosage on the shrinkage of foamed concrete with mass-equivalent cement replacement.

Foamed concrete typically exhibits higher drying shrinkage than conventional concrete due to its greater binder content and elevated initial moisture. The addition of highly reactive SCMs further influences the volumetric deformation of the cement paste. Figure 9 presents the 90-day drying shrinkage of foamed concrete under dry curing when OPC is partially replaced, by mass, with varying levels of SCMs. As depicted in Figure 9a, the drying shrinkage of foamed concrete increases progressively with higher levels of SF substitution. This phenomenon is primarily attributed to the mechanisms of cement hydration, wherein the evaporation of water from the paste generates volumetric contraction driven by surface tension within capillary pores. Owing to its extremely fine particle size, approximately 1/100 of that of ordinary Portland cement, SF effectively fills capillary voids and significantly refines the microstructure of the hardened paste, resulting in a denser pore network. However, this densification process restricts the volume available for moisture movement, thereby intensifying capillary pressure and exacerbating the propensity for shrinkage [63]. Furthermore, SF accelerates the rate of cement hydration, rapidly consuming internal moisture and intensifying early-age shrinkage [64]. A similar trend of increased shrinkage with higher SF content was observed in the study by Athraa H et al. [65], which was attributed to the high specific surface area and elevated water demand of SF. This indicates that the observed increase is due to microstructural reorganization rather than detrimental volumetric instability. In practical applications, the integration of fibers or the adoption of more effective curing regimes can compensate for the shrinkage effects induced by SF, thereby achieving a balance between mechanical enhancement and dimensional control.

Figure 9b illustrates that a moderate substitution of MP results in a reduction in drying shrinkage, with the minimum shrinkage observed at a replacement level of 3%. Beyond this point, shrinkage begins to increase with further substitution. This reduction at lower dosages is likely due to the delayed hydration kinetics of MP in comparison with Portland cement, which suppresses the early hydration heat peak and correspondingly mitigates early-age shrinkage [66]. The reactive components in MP, including SiO_2_, Al_2_O_3_, and CaO, participate in pozzolanic reactions with Ca(OH)_2_ released during cement hydration, generating additional C-S-H gel that contributes to long-term strength and dimensional stability. The fine fineness and high hydraulic reactivity of MP facilitate the development of a dense microstructure, which in turn helps mitigate shrinkage [67]. Nevertheless, excessive replacement with MP may hinder the early formation of hydration products, impeding the development of a robust internal matrix capable of resisting volumetric deformation. In such cases, the delayed establishment of structural integrity limits the material ability to counteract shrinkage-induced stresses effectively.

Figure 9c clearly demonstrates the shrinkage-reducing efficacy of MK. A steady decline in drying shrinkage is observed with increasing MK content, culminating in a reduction of approximately 40% at a 9% replacement level. Similar trends were observed by Athraa H et al. [65], who demonstrated that MK effectively contributes to pore size reduction and decreased water demand. The hydration products formed from MK, particularly C-A-S-H gel, exhibit higher density than conventional C-S-H, contributing to a more compact matrix and reduced pore volume. Consequently, the reduction in capillary porosity significantly limits moisture loss and the associated shrinkage. Moreover, MK exhibits moisture adsorption capabilities, which help regulate the internal water-to-binder ratio, maintaining the internal moisture environment during the early curing phase. This moisture buffering capacity slows the rate of evaporation and minimizes abrupt volume changes. By moderating the initial hydration kinetics of cement, MK promotes the gradual and controlled development of microstructure, resulting in improved dimensional stability over time.

## 4. Conclusions

This study initiated the hydrophobic surface modification of expanded perlite (EP) and subsequently developed a series of EP-aggregated foamed concrete mixtures. Ordinary Portland cement (OPC) was partially replaced with varying levels (3%, 6%, and 9%) of supplementary cementitious materials (SCMs), namely, silica fume (SF), mineral powder (MP), and metakaolin (MK). A comprehensive assessment of the mechanical and physical properties of the resulting composites was conducted through a series of material performance tests. The major conclusions drawn from the current investigation are summarized as follows:

1. The addition of SF and MP evidently enhanced the 28-day uniaxial compressive strength of EP-aggregated foamed concrete, likely due to the synergistic effects of their pozzolanic reactivity and micro-filler effects. MK showed a distinct optimal substitution level, beyond which further strength gains were not observed. This may be attributed to the formation of low-density calcium aluminosilicate hydrate phases and the reduction in the amount of reactive cement. Among all SCMs, MP yielded the highest strength gain at equal replacement levels, outperforming both SF and MK.
2. The thermal conductivity of EP-aggregated foamed concrete increased with SF and MP addition, particularly at higher substitution levels, which may be related to their ability to increase the solid phase proportion and bulk density of the concrete. Conversely, MK consistently reduced thermal conductivity, enhancing the material’s insulation performance.
3. EP-aggregated foamed concrete incorporating SF, MP, or MK exhibited reduced mass loss rates, improving durability as substitution levels increased. These phenomena suggested that SCMs likely enhanced the interfacial bonding and crack resistance, limiting microcrack propagation. The replacement of SF at 3% and 6% achieved the lowest mass loss rates, indicating an optimal range for durability enhancement. At the 9% level, MP provided the most notable improvement.
4. Increasing MP content led to higher volumetric water absorption, likely due to incomplete pore filling by MP and cement hydration products. In contrast, SF and MK reduced water absorption by decreasing pore connectivity, with MK showing the greatest effect.
5. Higher SF substitution increased the drying shrinkage of EP-aggregated foamed concrete, possibly due to accelerated hydration and pore densification. MP reduced shrinkage at moderate levels but increased it at 9%. MK consistently minimized shrinkage across all levels by regulating hydration and refining pore structure.

It is worth noting that this study mainly focused on macroscopic testing, and the substitution levels of SCMs were relatively low. Future work should emphasize microstructural analyses to better understand the mechanisms of SCMs in foamed concrete materials. In addition, exploring higher SCM replacement ratios is necessary to gain further insights into optimizing material performance and sustainability, thereby facilitating practical engineering applications.

## Figures and Tables

**Table 1:** Chemical composition and physical properties of MP, MK, and SF.

Supplementary Cementitious Materials	Chemical Compositions (w.-%)									Physical Properties		
	CaO	Al_2_O_3_	SO_3_	SiO_2_	Fe_2_O_3_	MgO	K_2_O	Na_2_O	Others	Specific Surface Area (m^2^/kg)	Loss on Ignition (%)	Average Particle Size (µm)
MP	40.15	11.95	1.80	34.58	2.52	5.77	0.48	0.36	2.39	439	<2.8	8–10
MK	0.20	42.60	0.05	48.70	4.90	0.28	0.46	0.26	2.55	>16,000	<0.75	2–4
SF	0.28	0.36	0.16	90.58	0.1	3.85	2.56	0.52	1.59	>15,000	2.10	0.1–0.3

**Table 2:** Mixture proportions of foamed concrete samples (unit: kg/m^3^).

Sample	OPC	MP	MK	SF	EP	Foaming Agent (%)	Water-To-Binder Ratio
A0	300	0	0	0	150	2.0	0.50
A0-MP-3%	291	9	0	0			
A0-MP-6%	282	18	0	0			
A0-MP-9%	273	27	0	0			
A0-MK-3%	291	0	9	0			
A0-MK-6%	282	0	18	0			
A0-MK-9%	273	0	27	0			
A0-SF-3%	291	0	0	9			
A0-SF-6%	282	0	0	18			
A0-SF-9%	273	0	0	27			

Note: The dosages of MP, MK, and SF represent their mass-based substitution ratios for a portion of cement. The foaming agent dosage is expressed as a percentage of the mass of the cement paste. For all mixtures, an equal volume of pre-prepared foam was incorporated to achieve the target density of the foamed concrete.

**Table 3:** The bulk density of samples.

Sample	Bulk Density (Unit: kg/m^3^)
A0	302 ± 10
A0-SF-3%	331 ± 10
A0-SF-6%	368 ± 10
A0-SF-9%	384 ± 10
A0-MP-3%	306 ± 10
A0-MP-6%	328 ± 10
A0-MP-9%	346 ± 10
A0-MK-3%	291 ± 10
A0-MK-6%	265 ± 10
A0-MK-9%	252 ± 10

## Data Availability

The original contributions presented in this study are included in the article. Further inquiries can be directed to the corresponding authors.
